# How do the media report cancer research? A study of the UK's BBC website

**DOI:** 10.1038/sj.bjc.6604531

**Published:** 2008-07-29

**Authors:** G Lewison, S Tootell, P Roe, R Sullivan

**Affiliations:** 1 School of Library, Archive and Information Studies, University College London, London WC1E 6BT, UK; 2 Evaluametrics Ltd, 25 Hanover Gardens, London SE11 5TN, UK; 3 Evaluametrics Ltd, 157 Verulam Road, St Albans AL3 4DW, UK; 4 Department of Social Policy, London School of Economics and Political Science, Houghton Street, London WC2A 2AE, UK

## Abstract

This study examined cancer research stories on the BBC web archive (July 1998–June 2006). There were about 260 BBC stories per year, of which about 170 were classed as relevant to reports of cancer research. The stories focused heavily on breast cancer, and over one-third of them mentioned this (compared with a cancer disease burden of 13%); the next most covered sites were lung and prostate cancers, although the former was much less mentioned than its cancer disease burden of almost 20% would have suggested. The focus of the stories was often on new or improved drugs or vaccines (20% of stories), with lifestyle choices (12%), genetic developments (9%), and food and drink (8%) also featuring fairly prominently. The BBC stories cited about 1380 research papers that could be identified as journal articles. About three-quarters of the cited papers were in the field of cancer. The papers of these authors came from over 60 countries, and 40% were from the United Kingdom and 36% from the United States. UK cancer research was heavily overcited, by about 6:1, relative to its presence in world oncology research and US research was cited about in proportion. That of most other countries, especially Japan, Germany, and Austria, was relatively undercited. These cited papers also acknowledged more funding bodies. Most of the BBC stories were put in context by external commentators, of whom the large majority was from the UK's cancer research charities.

UK cancer research has enjoyed substantial sociopolitical focus over the last 8 years with the merger of the two largest charities and the creation of the National Cancer Research Institute. Such focus has inevitably given rise to a greater need to demonstrate the impact of national research expenditure and to influence health-care policy (Glass, 2002). Mass media are the nexus between public and policy agenda and are highly influential in shaping discourses about health and research. The way in which news media affect the public is complex and diverse. Recognised effects include informing audiences (Rees and Bath, 2000), agenda-setting, framing (Passalacqua *et al*, 2004) and persuading (Iyengar, 1997). There is now a substantial corpus of literature demonstrating the impact of media on shaping public opinion towards countries’ health-care systems (Benelli, 2003; Collins *et al*, 2006), and how newspapers targeted at particular ethnic groups can vary in their approach (Hoffman-Goetz and Friedman, 2005). In addition, there have been studies of how health-related issues have been portrayed in the media around particular diseases (Brown *et al*, 2001), the uptake of health care (Mintzes *et al*, 2003) and screening for cancer (Jones, 2004; Schroy *et al*, 2008), and pharmaceutical coverage (Cassels *et al*, 2003), particularly in certain media-conscious countries such as Australia, Canada, the United Kingdom and the United States. However, the mass media have also given misleading information about the supposedly beneficial effects of complementary and alternative therapies (Milazzo and Ernst, 2006; Weeks *et al*, 2007).

The nature and impact of science in the media have also become a major policy concern (Conduit, 2001). Commentators on this subject have given vent to a range of complaints, such as the accuracy of media reporting (MacDonald and Hoffman-Goetz, 2002; MacKenzie *et al*, 2007), the pressure of commercialisation and the challenge of media hype (Ooi and Chapman, 2003; Caulfield, 2004). However, the impact of disease-specific research on the media remains a relatively underdeveloped and understudied area. This study set out to describe in a quantitative manner the impact of cancer research on a major conduit of research stories – the BBC news website (http://www.bbc.co.uk) – which is accessed by some 52% of the UK online universe, some 13.2 million people annually. This source is also used for both the national and international press, TV, and radio stories, and thus provides an ideal surrogate for the determination of overall media impact. The goals of this study were broadly to map the impact of research funding organisations and their commentators on reported cancer research stories, to determine the extent to which the media reporting of cancer research is ‘balanced’ in terms of its site-specific coverage and domains of research, and finally the degree to which media reports of cancer research by the BBC reflect the international impact and indeed whether such reporting in turn influences the citation of papers.

## Methodology

The search of the BBC archive was limited to the health section and to the 8-year period from July 1998 to June 2006. The headline, date and abstract of the stories were copied from the BBC website to a spreadsheet, and they were each read through (by GL and ST). They were first coded for relevance (3 for being relevant because they cited research; 2 for being partly relevant, usually because they reported future research or some survey of patient attitudes or experiences; 1 for not relevant – often a report of an individual case). For example, stories with headlines such as ‘Vitamin D can lower cancer risk’ or ‘Virus clue to cervical cancer jab’ were coded 3, and those headed by ‘Boys less likely to eat healthily’ or ‘Ethnic minorities less breast aware’ were coded 2.

Selected data (cancer site – e.g., breast or lung, scientists involved, their institution(s), the journal in which any cited paper was published, and details of any commentators) were then extracted and recorded on the spreadsheet for each story. The percentages of BBC stories focusing on different cancer sites were compared with the UK's burden of disease from these particular cancers, measured in Disability Adjusted Life Years (DALYs) (Murray and Lopez, 1996), as given by the World Health Organization for 2002, and relative to the burden from all cancer – this gives a fairer impression of the effect of different cancer types on society than the numbers of deaths. The stories were also coded for the basis of the work being reported, namely drug-related, environmental, food and drink, genetics, job, lifestyle, as 1 or 0 in each of six columns on the spreadsheet.

If a story cited one (or more) research paper, then the bibliographic details of these were sought. They could usually be identified readily because the name of the journal was given, but occasionally this turned out to be given incorrectly. Some conference presentations could be identified with meeting abstracts in relevant journals, although for others there did not appear to be a corresponding publication. The addresses of all the authors were also carefully recorded, with at least three elements present: the institution name, the city name (and region/state and postcode, if present), and the country. These addresses could then be analysed by means of special macros to reveal both integer and fractional counts of countries. For example, a paper with one UK and two US addresses would be counted as unity for both countries on an integer count basis, but 0.33 and 0.67, respectively, on a fractional count basis.

The research level (RL) of the cited papers was determined to see if the BBC stories primarily covered clinical work, as might have been expected, or sampled fairly the whole range of cancer research as reported in the peer-reviewed serial literature. The research level was calculated on the basis of the journal in which the papers were published as a decimal number between 1 and 4, where 1=clinical observation and 4=basic research. This was determined from the titles of the papers appearing in the given journal that had a biomedical word in their addresses (Lewison and Paraje, 2004). Over 100 ‘clinical’ title words and a similar number of ‘basic’ words were used to determine if a journal paper was clinical, basic, or both: clinical papers were given an RL of unity, basic papers an RL of 4, and ‘both’ papers an RL of 2.5. From these values, it was possible to calculate the mean RL for papers in the journal. Some examples of leading journals with their RLs are given in Table 1.

The potential citation impact (PCI) of the cited papers was also based on their journal and was determined as the mean number of citations received by papers in the journal in the year of publication and four subsequent years. However, as this gives values that do not correspond well to the subjective views of both researchers and administrators (Lewison, 1995, 1998) on the relative importance of papers in different journals (which are in the range 1–5 or 6), a logarithmic function (LOG) was also calculated: 1+2log_10_(1+PCI), whose values range from 1 for ‘ordinary’ journals up to 5 for ‘top’ journals such as *Nature*. Some examples are shown in Table 1.

The actual citation impact (ACI) of those cited papers published in 1998–2001 with addresses only in the United Kingdom, or in the United States (in practice, just over 300 papers) was determined by the counting of citations to them in their year of publication and 4 subsequent years. The address details of all the citing papers were also determined. This was done to gauge whether citation by the BBC influenced the papers’ impact on other scientists.

The individual cited papers were also looked up to record their funding acknowledgements. Each such acknowledgement was recorded with four codes: a trigraph to identify the individual organisation (e.g., MRC=Medical Research Council), a letter to denote the type of funding (I=intramural, E=extramural, P=personal, and K=in kind), a digraph to denote the organisation's category (e.g., CH=collecting charity, FO=endowed foundation, GA=government agency, and IP=pharma industry), and another digraph to denote the country of the organisation, taken from the International Standards Organization list. A few funding bodies were European, and coded EU; and some were international and coded XN.

All of these parameters – geography, RL, PCI, ACI, and funding – were also compared with the corresponding values (both means and distributions) for world oncology research papers for relevant years so as to normalise the results and show whether the papers cited by the BBC were, or were not, unbiased samples from the larger population. The world oncology research files were derived from the Science Citation Index by means of a ‘filter’ (Cambrosio *et al*, 2006) that was based on specialist cancer journals and title keywords; they contained details of about 35 000 papers per year.

Many of the BBC stories attempted to put the research news in context with comments from external experts. The names of these people, and their organisations – usually cancer research funders – were recorded. Some unification of the organisation names was needed, and their percentage presence among the commentators was compared with their presence among the funders of UK cancer research in 2000–2001 (Webster *et al*, 2003) so as to normalise the results and to see if the BBC ‘experts’ were representative of the cancer research funding community in the United Kingdom.

## Results

### Numbers of stories and cancer sites

**Figure 1:**
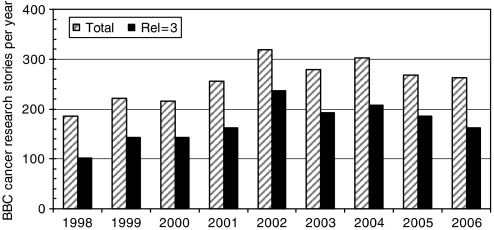
Annual numbers of BBC stories on ‘cancer+research’ or ‘cancer+study’ from July 1998 to June 2006. Stories of high relevance (rel=3) are shown in black.

Figure 1 shows the numbers of BBC cancer research stories in each of the 9 years of the study. Relevant stories categorised as ‘3’ are shown in black. The number of stories reached a peak in 2002 and has since declined.

**Figure 2:**
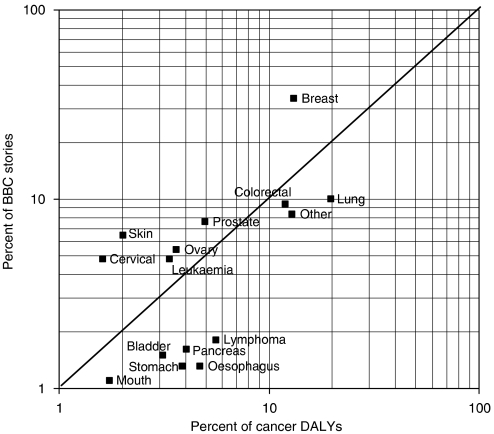
Correlation between the relative burden of different cancers (DALYs, 2002, WHO estimate) and the numbers of BBC stories mentioning the different cancer sites, 1998–2006.

Of the relevant stories, some mentioned several cancer sites, and others did not mention any particular site. What was clear was that breast cancer dominated, with over one-third of all stories (where one or more sites were mentioned) referring to it. Lung cancer (10%) came a rather poor second, followed by prostate (8%) and skin cancer (6%). Figure 2 shows a plot of percentage of mentions of different cancer sites against percentage of total cancer DALYs for the UK for these sites. This plot makes it clear that cancers of breast, cervix, and skin are overreported in relation to the burden they cause. Of the major cancer types, the biggest ‘deficiency’ is in lung cancer, where there are far fewer stories than the burden of this disease would suggest. It causes 20% of all cancer DALYs, but is only mentioned in 10% of the BBC stories.

### Story features

**Figure 3:**
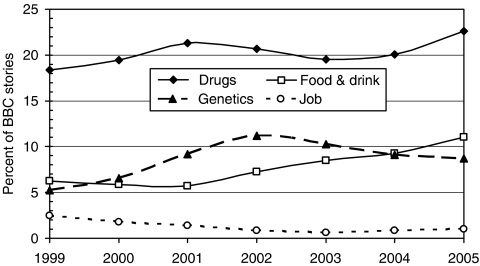
Variation of relative presence of four types of story among BBC cancer research stories of 3-year moving averages. (Environmental and lifestyle stories were relatively constant at 5 and 12%, respectively.)

New and improved drugs (and a few about vaccines) are the dominant type of story, followed by ones on lifestyle (particularly smoking and sunbathing), genetics, and food and drink (including dietary supplements such as vitamins) (Figure 3). Coverage of new drugs peaked in 2001, and then declined; the recent rise is largely due to stories about whether herceptin should be prescribed on the NHS for the early-stage breast cancer. Genetics stories rose to a peak in 2002 and have also declined somewhat. Meanwhile, stories about food and drink have steadily increased in presence; this may also reflect a generally increasing interest in food, including school dinners. But stories about occupational risks, never numerous, appear to have declined steadily; this probably reflects the continuing decline of ‘dirty’ industries in western Europe and North America and their replacement with relatively safer service jobs.

### Cancer research papers cited by the BBC stories: journals and geography

About two-thirds of the BBC stories reported cancer research advances, and some stories cited more than one research paper. In total, there were 1394 cited research papers in 253 different journals that could be identified from the information given in the story (37 papers could not be identified). Some of the leading journals are listed in Table 1, and the cancer journals are shown in bold: altogether, they account for 42 of the total, and for 443 papers, or 32%. (This is fairly typical: in most biomedical sub-fields, only about one-third of the papers are in specialist journals; Lewison, 1996.)

**Figure 4:**
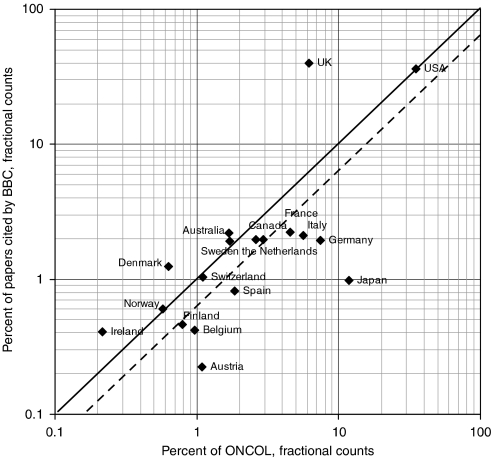
Comparison between percentage presence (fractional counts) of 18 countries in papers cited by BBC stories and their presence in oncology research (2000–2004). Solid diagonal shows equal relationship; dashed diagonal shows relationship expected when UK contributions to papers are removed from the international pool.

Of the cited papers, 1036 (76% of the identified papers) fell within the oncology subfield (ONCOL), as defined by the Cancer Research UK oncology filter, described above. The cited papers included authors from 62 different countries, but the United Kingdom and the United States each had a large proportion of the total on both a fractional and an integer count bases (Figure 4).

Figure 4 shows the percentage presence of 18 leading countries in the papers cited by the BBC stories plotted against their presence in oncology research. Although it looks as if the stories were dominated by research from just two countries, the US papers were cited closely in proportion to their presence in oncology research in the 5 years, 2000–2004, and it is just the UK papers that were, not altogether surprisingly, overcited. Indeed a very similar result was found for biomedical research papers cited in UK newspaper stories in 2001 (Lewison, 2002). The papers of two European countries (Denmark and Ireland) were also overcited, but not to the same extent. Research carried out in most European countries was undercited, especially Germany and Austria, as was that of Japan. If UK contributions both to papers cited by the BBC and to oncology are removed, a few more countries (Canada, Netherlands, and Switzerland) now appear to be slightly ‘above the line’.

The difference between the observed presence (number of papers and fractional counts) and that expected from the oncology file can be determined as statistically significant or not. For Ireland, the difference is not significant (*P*∼10%), but for Denmark it is *P*∼0.2%, and for the larger continental countries, the (negative) difference is highly significant (*P*<0.01%).

### Cancer research papers cited by the BBC stories: RL and PCI

**Figure 5:**
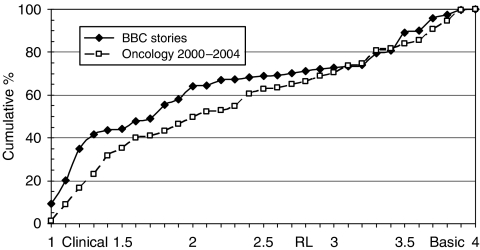
Cumulative distribution of research levels (1=clinical and 4=basic) of papers cited by BBC (solid line) compared with world oncology papers, 2000–2004 (dashed line).

**Figure 6:**
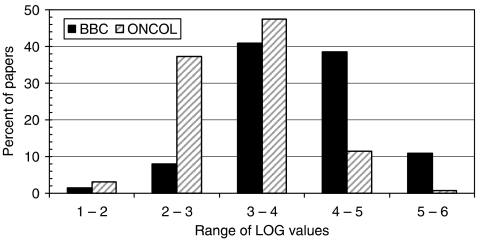
Distribution of LOG function for potential citation impact for papers cited by the BBC (black bars) and world oncology papers, 2000–2004 (striped bars).

In this section, a comparison is made between the distribution of the RLs of the papers cited in BBC stories and those of oncology research papers from 2000–2004. Similarly, the distributions of a log function (LOG) of PCI (based on the journals in which the papers were published) have also been compared. For RL, the comparison takes the form of cumulative distribution curves (see Figure 5); for LOG, distributions are shown as a chart in Figure 6, although mean values have also been calculated and are shown in Table 2.

Papers cited by the BBC stories are, on average, more clinical than the oncology papers (Figure 5) and they are also in much more highly cited journals (Figure 6). The mean values of PCI, and of its corresponding log function (LOG), have been rising slowly with time, but they are far below the mean value for the papers cited by the BBC stories. By way of comparison, a mean RL of 2.28 corresponds to *Journal of Cancer Research and Clinical Oncology*; a mean RL of 2.06 corresponds to *Neoplasma*. A mean PCI of 13.9 corresponds to *British Journal of Cancer* or to *Journal of Mammary Gland Biology and Neoplasia*, and a mean PCI of 34 corresponds to *Seminars in Cancer Biology*.

### ACI of papers cited in BBC stories

The mass media are not only seen, heard, and read by the public, but they also attract the attention of the research community. For example, it was found that *The New York Times* had a marked effect on the frequency with which the research articles that it wrote about were cited from a comparison of the numbers of citations to articles in the *New England Journal of Medicine* published before, during, and after a 3-month strike at that newspaper (Phillips *et al*, 1991). We hypothesised that coverage by the BBC might have a similar effect. However, in the absence of the unique occurrence of the strike (when a paper of record was prepared but not distributed), it is more difficult to normalise and determine the counter-factual situation.

We applied two tests to see whether citations by the BBC made a difference to the impact of the papers on the research community. First, we compared the mean actual citation counts with samples of oncology papers with only UK authors, or only US authors, from the year 2000 with their mean potential citation counts, that is, the expected citation scores if they were typical of papers published in the same journals. We then compared the same indicators for two sets of BBC-cited papers, again with just UK authors or just US ones. (The reason for the geographical exclusiveness is that it is known that multinational papers tend to be published in higher impact journals and to receive more citations, but the effect is somewhat dependent on the numbers of countries involved and their identity, and also on the numbers of funding sources acknowledged; Narin *et al*, 1991; Lewison, 2003.) The results are shown in Table 3.

Both sets of oncology papers received fewer citations, on average, than might have been expected from the journals in which they were published, but both sets of papers cited by the BBC received more citations than expected. This suggests either that the BBC reporters were prescient in selecting the papers that would be highly cited or that they influenced the process – perhaps the latter explanation is more likely, as BBC coverage may well have led to stories in the newspapers as well and thus to greater publicity for the papers, and not only in the UK. They may also have been pre-selected by the journal staff, who often send out press notices to draw attention to papers thought to be of particular interest and significance.

The second test was to see whether citations in BBC stories had a disproportionate influence on UK scientists, as measured by the propensity of these papers to have a higher percentage of their subsequent citations from the United Kingdom. For this purpose, we downloaded bibliographic details of all the citing papers from the above table (namely, 5466+11 007+3599+8950=29 022) and analysed them geographically so as to reveal the (fractional count) contributions of the different citing countries. The results are shown in Table 4.

Data for Canadian citing authors have been provided to show that for a third country, the percentages of their citations are almost constant. The column head ‘UK, %’ shows that UK authors were more frequent citers of both groups of papers mentioned in the BBC stories, by 28.2/24.1 or by +17% for the UK papers and by 5.9/5.0 or by +18% for the US ones. The effect is not large, but the differences between the observed and expected values on the null hypothesis are statistically significant (*P*=0.003% for the US papers, and even less for the UK ones). The authors from US were less likely to cite the UK articles in BBC stories, but they were more likely to cite the US papers, which probably also received coverage in their own country's media.

### The funding of the cited research papers

**Figure 7:**
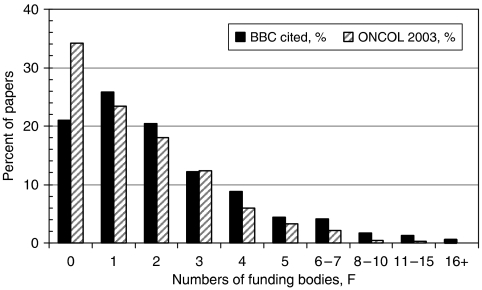
Distribution of numbers of funding bodies acknowledged on papers cited by BBC cancer stories, 1998–2006, and on a sample of 2115 oncology papers from 2003.

Altogether, funding acknowledgements were recorded for 1385 papers, of which 594 had an address in the United Kingdom and 647 had an address in the United States. Of these 1385 papers, 290 or 21% had no recorded funding, and the research was probably supported by general university funds or those of the relevant hospital or state health service based on the presence of university or hospital addresses. The others had one or more funding acknowledgements, the maximum number being 39 for two papers. Figure 7 shows the distribution of the numbers of funders, with, for comparison, the corresponding percentages found for a large structured sample of oncology papers from 2003.

It is clear that the papers cited by the BBC stories have more funding acknowledgements and that, in particular, relatively few have ‘no’ funding – 21% compared with 34% for the sample of oncology papers. An analysis was then made of the sector of the funding bodies for UK and US papers. For this purpose, funders were divided into four main sectors, namely national government (including, local and regional authorities), national private non-profit, industrial, and international. There were also other funders, public and PNP sector organizations from third countries. The sectoral analysis is shown in Table 5.

As would be expected from Figure 7, there are many fewer ‘unfunded’ papers than expected for both the United Kingdom and the United States. In the United Kingdom, funding is dominated by the private non-profit sector, but in the United States it is led by the government sector, with much support coming from the National Cancer Institute (NCI) and from the National Institutes of Health (NIH) more generally. Industry funds about one-sixth of the papers cited by the BBC in both countries. The international funding is primarily from the European Commission, which was acknowledged on 48 of the papers cited by the BBC, of which 35 were from the United Kingdom and 9 from the United States (all, of course, co-authored with a European Union Member State). By contrast, the World Health Organization (WHO) was acknowledged on only twelve papers cited by the BBC, of which four were from the United Kingdom and six from the United States, and two from both countries.

### Commentators on the research findings

**Figure 8:**
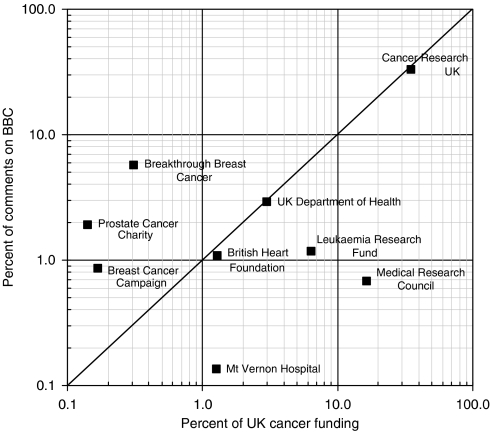
A plot of percentage presence of UK cancer funding organisations as commentators on BBC stories of cancer research, 1998–2006, against their contribution to UK cancer research funding, 2000–2001.

A notable feature of most of the BBC stories was that external experts were asked to comment on the significance of the results, and what they might mean for the treatment of cancer patients. Altogether, there were as many as 724 different commentators over the 8-year period, and they made a total of 1842 comments on 1272 stories, with an average of 1.45 quotes on each story where a commentator was quoted. Of the 12 most frequent commentators (one of whom was RS) with 19 or more appearances, no fewer than 10 were from Cancer Research UK, and the other 2 were also from cancer research charities (Breakthrough Breast Cancer and the Prostate Cancer Charity). Public sector commentators were relatively rare. They included Julietta Patnick of the NHS Cancer Screening Programme, Mike Richards, the National Cancer Director, and also 10 Members of Parliament. The Medical Research Council (MRC) commented only on 15 stories, just over 1%. Figure 8 shows that there is no correlation at all between support for cancer research in the United Kingdom and invitations to comment, except for Cancer Research UK and the Department of Health (UK), for which the invitations are in fair proportion. Representatives of the Wellcome Trust, which, despite formally eschewing the funding of clinical cancer research in the United Kingdom (Wellcome Trust, 2008), funded about 7% of UK cancer research in 2000–2001, did not comment at all, and two cancer charities based outside London, the Yorkshire Research Campaign in Harrogate and the Association for International Cancer Research in Fife, Scotland (both funding 2.3% of UK cancer in those years) only commented once and twice, respectively.

These findings suggest that the BBC (in common with other news media) see it as highly desirable to put new research findings in context. Most commentators were enthusiastic and viewed the research as very useful, but they counselled strongly against premature optimism and stressed that more research work was needed, and more time, before patients were likely to benefit. The frequency with which the BBC reporters (nearly all anonymous, unlike in the newspapers) turned to the cancer charities in London for interpretation of the research news suggests that this path has become well-worn and, in turn, that the charities have become adept at presenting themselves to the public even if only in the reflected glory of research done by others. Although the MRC and the Wellcome Trust are active in cancer research, one would not realise this from the BBC stories.

## Discussion

The BBC archive, which closely approximates to the items broadcast on radio and television, is a fecund source of bibliometric data (Ghosh, 2007). It provides a new perspective on biomedical research and helps reveal its likely impact on the wider public. In this study, we have found consistently high levels of BBC cancer research stories. The impetus of charitable mergers and additional government support to cancer research appears to have been reflected in the absolute numbers of cancer research stories reaching a peak in 2002. However, the wave of activity appears to have hit a high tide mark and has been settling back down over the last 3 years to pre-2000 levels.

We have found, relative to the DALY impact, a strong focus on breast cancer in these stories. Within the cancer field, breast cancer is acknowledged to have strong personal identity and worldwide advocacy stemming from activism in the late 1980s and a research track record of delivering new and effective management in the form of greater understanding of pathogenesis and risk factors (e.g., Million Women Study/Hormone Replacement Therapy) through surgical techniques (Sentinel Node Biopsy) and drugs (e.g., the wide range of endocrine therapies). This self-reinforcing cycle has filtered through to generate huge media exposure, when compared with other site-specific cancers. This has been a mixed blessing. Although this clearly provides greater breast awareness and potentially increases charitable giving to breast cancer (although there is no direct evidence that this happens), we know that women greatly overestimate their lifetime risk of breast cancer (Pohls *et al*, 2004). Why? The plausible hypothesis is that the greater exposure of women to breast cancer stories, particularly those associated with risk factors, leads to a resetting of the cultural milieu in which women estimate their risk.

A number of site-specific cancers fare poorly in their media exposure – lung, upper GI tract, and so on. For the majority of these cancers (lymphoma being an exception to this observation), progress in controlling and curing them has been glacial. Mortality is high and survival poor. Thus, a lack of year-on-year treatment progress coupled to poor outcomes has inevitably led to poor media exposure. As art reflects society, so the media are reflecting progress – those cancers for which we are ahead of the curve (e.g., breast), those on the curve (e.g., colorectal), and those that are behind (e.g., upper GI tract and lung). Media trends in coverage of site-specific cancers over time appear from our data to be an additional tool to inform policymakers not only of active/successful research domains but also of those that have developed strong interest groups (Howlett and Ramesh, 2003).

In a typical year, there will be about 240 BBC stories on cancer research, of which about 160 will cite research papers. The main focus is on new and improved drugs that could be used to treat cancer. Drugs dominate research reports and the trend is increasing. Such stories overwhelmingly emphasise breakthroughs, and, as previously reported, do a poor job in reflecting the relative contribution of the drug in question and indeed the multitude of giants on whose shoulders such new technologies have come into being (SIRC, 2001). Again the media are reflecting the dominant research paradigm in cancer – that of drug discovery and development – and providing a surrogate marker not only for the activity levels of research in this area (which are very high compared with other domains, e.g., prevention) but also the proactive sociopolitical advocacy of the organisational structures in which the majority of researchers are based, namely the pharmaceutical and biotechnology industries. The economic model of these organisations demands media communication as a tool for leveraging funding and/or influencing the stock market and shareholders. Such proactivity leads to hype and a lack of relativism in the communication by the media of cancer research stories, a constant theme in health literacy and public policy (Hayes *et al*, 2007). Should policymakers be concerned? In sociological terms they should be, because hype invariably leads to unmet public expectations; the knock-on effect is a backlash against such activities (or rather the funding of them) and a decline in trust (Caulfield, 2004). The latter, considering the current issues of trust around commercially driven research anyway, is particularly damaging to the public credibility of science (DeAngelis, 2000). Despite the fact that the story types probably reflect the relative research activity levels in these domains, there is a clear need to promote more balance with non-drug cancer research stories.

The papers cited by the BBC, numbering about 1380, are overwhelmingly from the United Kingdom (which are overcited by the BBC by a factor of about 6) and from the United States, which together comprise about 84% of the total. Although they tend to be rather more clinical than the average for cancer research papers overall, basic research is by no means neglected. They are taken from a very wide range of journals (over 250), but three UK journals, namely *The Lancet*, *British Journal of Cancer*, and *British Medical Journal*, dominate. On average, the cited journals have high impact factors – the mean is more than twice that for cancer research papers overall. What these findings do suggest is that media reporting of cancer research by the BBC is, relative to global cancer research activity and outputs (publications), narrow. One argument against this might suggest that important research is (a) only published in the lingua franca of science, that is English; (b) is thus dominated by native English speaking countries, that is the United Kingdom and the United States; and (c) will inevitably be published in the higher impact journals. The problem is that a high impact journal will consequently produce research that has a high impact on the public, but the few qualitative studies in science have actually found a very poor correlation between the initial impact of a paper (as defined by the journal) and its eventual impact. The other major issue is the perceptual bias that an undue focus on a very selective group of journals and countries has on the public's view of cancer research. Such situations lend too easily to the creation of a hegemony where biases perpetuate themselves and create a public frame of reference around cancer research that has a false logic and consciousness (Sallach, 1974).

Finally, we have attempted to address what impact, if any, the reporting of cancer stories by the BBC has had on the research community. Our data suggest a possible influence, but this study cannot rule out the possibility that the BBC news team consistently pick higher impact papers (although this is unlikely from other studies). How such stories impact the general public's perception of cancer research in all its permutations is a logical question that can be built upon the data presented. Similarly, the variability in which cancer research is presented through the media in different European Member States and more widely would say much about the different cultural perceptions towards this particular biomedical research domain. Cancer research does not have a well-developed ‘third culture’ (direct researcher-to-public connection), and hence is increasingly reliant on the diverse media for informing and engaging the public. If the aim is to achieve a ‘public understanding of cancer’, then we need to ask how this current interface works and whether it is achieving the aspirations of both the public and the research community.

## Figures and Tables

**Table 1:** List of some leading journals cited in BBC cancer stories, with the number (*N*) and percentage of papers cited, their research level in 2000–2004 (RL1=clinical; RL4=basic), PCI over 5 years and log value (LOG)

**Journal**	**N**	**%**	**RL**	**PCI**	**LOG**
*Nature*	55	3.9	3.48	100.6	5.0
*New England Journal of Medicine*	53	3.8	1.16	95.3	5.0
***Journal of the National Cancer Institute***	71	5.1	1.79	52.1	4.4
*JAMA* – *Journal of the American Medical Association*	53	3.8	1.07	49.9	4.4
*Proceedings of the National Academy of Sciences of the United States of America*	50	3.6	3.70	43.2	4.3
***Cancer Research***	43	3.1	3.29	34.6	4.1
*Lancet*	155	11.1	1.24	29.7	4.0
*British Medical Journal*	102	7.3	1.04	14.4	3.4
***British Journal of Cancer***	107	7.7	2.04	14.0	3.3
***European Journal of Cancer***	24	1.7	1.63	9.7	3.1
*Journal of Epidemiology and Community Health*	11	0.8	1.07	7.9	2.9

LOG=logarithmic function; PCI=potential citation impact; RL=research level.

Specialist cancer journals shown in bold.

**Table 2:** Mean values of RL, of PCI and of a log function of PCI [1+2log_10_(1+PCI)] (LOG) for oncology papers from years 2000–2004 (ONCOL), and for the papers cited by the BBC cancer stories (BBC)

**ONCOL**	**2000**	**2001**	**2002**	**2003**	**2004**	**BBC**
Mean RL	2.26	2.28	2.27	2.28	2.29	2.06
Mean PCI	13.4	13.7	13.9	13.9	14.3	34.0
Mean LOG	3.07	3.09	3.11	3.11	3.14	3.79

LOG=logarithmic function; PCI=potential citation impact; RL=research level.

**Table 3:** Comparisons of mean actual and potential citation impact over five years of four groups of papers: UK- and US-authored, and in oncology research (ONCOL, 2000) and cited by the BBC (1998–2001)

**Group**	**Papers**	**Cites**	**Mean ACI**	**Mean PCI**	**Ratio**
UK ONCOL	441	5466	12.4	14.8	0.83
US ONCOL	674	11 007	16.3	18.1	0.90
UK BBC	154	3599	23.4	20.2	1.16
US BBC	152	8950	58.9	46.7	1.26

ACI=actual citation impact; PCI=potential citation impact.

**Table 4:** Geographical analysis (Canada, the United Kingdom, and the United States; fractional counts) of papers citing to four groups of papers (see Table)

**Group**	**Cites**	**CA cites**	**CA, %**	**UK cites**	**UK, %**	**US cites**	**US, %**
UK ONCOL	5466	150	2.7	1317	24.1	1670	30.6
US ONCOL	11007	325	2.9	553	5.0	5815	52.8
UK BBC	3599	98	2.7	1014	28.2	1033	28.7
US BBC	8950	252	2.8	530	5.9	4990	55.8

**Table 5:** Sectoral analysis of numbers (*N*) and percent of different funding bodies acknowledged on UK and US papers cited in BBC cancer research stories with, for comparison, UK oncology papers from 2000–2001 (ONC, *n*=5385) and US oncology papers from 2003 (ONC, *n*=472)

	**UK, *N***	**UK, %**	**ONC %**	**US, *N***	**US, %**	**ONC %**
All papers	594	100	100	647	100.0	100
Government	170	28.6	18.3	396	61.2	57.6
Private non-profit	344	57.9	43.3	288	44.5	36.7
Industry	95	16.0	14.3	122	18.9	11.7
International	48	8.1	5.6	20	3.1	1.1
Other	132	22.2	22.8	145	22.4	n.a.
None	126	21.2	35.1	102	15.8	22.0
